# Preliminary Assessment of a Flexible Multi-Sensor Wearable System Based on Fiber Bragg Gratings for Respiratory Monitoring of Hemiplegic Patients

**DOI:** 10.3390/ijerph192013525

**Published:** 2022-10-19

**Authors:** Martina Zaltieri, Carlo Massaroni, Joshua Di Tocco, Marco Bravi, Michelangelo Morrone, Silvia Sterzi, Michele Arturo Caponero, Emiliano Schena, Daniela Lo Presti

**Affiliations:** 1 Unit of Measurements and Biomedical Instrumentation, Università Campus Bio-Medico di Roma, 00128 Rome, Italy; 2 Unit of Physical and Rehabilitative Medicine, Università Campus Bio-Medico di Roma, 00128 Rome, Italy; 3 ENEA Research Center of Frascati, 00044 Rome, Italy

**Keywords:** fiber Bragg gratings, respiratory monitoring, respiratory rate, chest wall asynchronies, hemiplegic patients, rehabilitation, wearable system

## Abstract

Respiratory diseases are common in post-stroke hemiplegic patients and represent a major social problem as they worsen the quality of life and reduce the life span. As a consequence, being able to monitor respiratory parameters such as the respiratory rate (RR) and assess the presence of respiratory asynchronies could be of paramount importance to define hemiplegics’ health status. Moreover, RR is a useful parameter to investigate the level of fatigue and distress that these patients undergo during rehabilitation processes. Although motion capture systems and flowmeters are the leading instruments for respiratory pattern evaluation, smart wearable systems are gaining ever more acceptance since they allow continuous monitoring by detecting chest wall breathing displacements, ensuring reduced costs and no need for dedicated spaces. Among other sensing technologies, fiber Bragg grating (FBG) sensors have emerged thanks to their high sensitivity to strain, lightness, and multiplexing capability. In this work, a wearable system composed of four flexible dumbbell-shaped sensing modules is proposed for respiratory monitoring in hemiplegic patients. The system is light and easy to wear and can be adapted to any anthropometry thanks to the modular anchoring system. Its feasibility assessment in RR evaluation was performed on seven hemiplegic volunteers in eupnea and tachypnea breathing conditions. In addition, an explorative investigation was conducted to assess the system’s ability to detect asynchronies between torso compartments. The good results suggest that this device could be a useful instrument to support clinicians and operators in hemiplegic patients’ management.

## 1. Introduction

In 80% of cases, stroke events result in the development of unilateral paresis, with hemiplegia being the most severe form [1,2]. Hemiplegia is a condition of partial or complete paralysis of an entire side of the body that seriously compromises the natural physical motility of the affected side. In particular, changes in the functionality of the trunk musculature and eventual diaphragmatic impairments often result in breathing dysfunctions and altered respiratory patterns, which worsen the patient’s quality of life and reduce the life span [3]. In the literature, various alterations in the ventilatory pattern of hemiplegic patients have been reported. Several studies proved that rib cage muscle weakness would cause, in the most severe subjects, paradoxical motion of the chest wall paretic side, thus resulting in breathing discoordination and asynchronous respiratory movements between torso compartments [4,5,6,7]. Breathing asynchronies are largely known in the literature and their effect on respiratory signals has been investigated. In fact, breathing discoordination between compartments can cause a phase shift between the related respiratory signals—that is, the more pronounced it is, the more severe the asynchrony [4,5,6,7]. The phase shift is commonly quantified by means of the phase angle (*Φ*) retrieved via the loop technique using Lissajous figures [8,9,10].

Other effects caused by respiratory muscle impairment are the reduction of vital and residual lung capacity, decrease in expiratory reserve volume, and a significant reduction in both inspiratory and expiratory volumes, leading to a constant sense of breath shortness and augmented respiratory rate (RR) [3,11]. In particular, a 25% increase in RR was observed in patients more than three years after stroke diagnosis compared to those with a more recent diagnosis [12], suggesting that respiratory muscles’ performance decreases in time with the consequent worsening of breathing efficiency. In fact, the more severe the disabling condition, the more serious the respiratory alteration [13]. However, conventional rehabilitation programs promote respiratory function recovery [14].

In this context, being able to determine patients’ RR could be of great help to monitor the subjects’ general state of heath and to understand the evolution of their condition in time. Moreover, since RR is strictly related to a state of mental and physical distress [15], monitoring its variation during rehabilitation sessions may allow the inspection of the state of fatigue experienced by patients. In addition, investigating the presence of breathing asymmetries between respiratory compartments may add information about the level of chest wall muscular impairment [12].

Motion capture (MoCap) systems are, to date, the leading and most reliable instruments to evaluate the respiratory biomechanics of patients [16]. However, the need for equipped spaces and qualified operators, the application of photo-reflective markers to the subjects’ torso, as well as the high costs of infrared cameras and dedicated software have limited the usage of this technology to hospital or ambulatory scenarios. Flowmeters [17] are also typically exploited, but are not free of drawbacks. Indeed, their usage implies the application of bulky breathing masks, limiting the user’s field of vision, or uncomfortable mouthpieces, which may strain the mouth musculature during long experimental sessions. A viable alternative to these limitations is represented by smart wearable systems. Garments and accessories (such as elastic bands, belts, etc.) embedding various sensing technologies have gained even more popularity as they permit unobtrusive and continuous monitoring, ensuring no weight, bulkiness, or motion limitation for the user. These systems are typically equipped with strain sensors (e.g., resistive [18,19,20,21], capacitive [22,23], inductive [24,25], or fiber Bragg grating (FBG) sensors [26,27,28,29,30,31,32,33]), allowing respiratory monitoring via the detection of breathing-related chest wall deformations [34].

In the last few years, FBGs have gained broad acceptance for RR detection [35]. Their lightness, flexibility, and reduced size allow for easy integration into textiles or polymeric matrices, while the multiplexing capability permits multiple sensors to be housed within the same fiber, ensuring limited encumbrance [36]. Such systems have been largely applied in several scenarios (e.g., clinical, sports, and working environments) [26,27,28,29,30,31,32,33], showing good performance in healthy subjects. Nevertheless, monitoring hemiplegics’ respiratory patterns, often characterized by paradoxical chest wall movements, may represent a major challenge that could worsen the efficiency of such devices.

For the first time, in this work, we assess the ability of an FBG-based wearable system to monitor RR in a population of hemiplegic patients. Moreover, an explorative investigation is carried out into the system’s ability to detect the presence of respiratory asynchronies. The exploited wearable device (whose manufacture, metrological characterization, and first assessment in a working environment have been presented in [37]) comes as two elastic bands intended to be worn around the chest and abdomen, each of which is equipped with a sensing element. This design ensures data collection in four areas of the torso (i.e., right and left thorax, and right and left abdomen), which can be beneficial given the limitations in chest wall movements that hemiplegics may experience. The device was tested against a reference instrument (i.e., MoCap) on seven hemiplegic volunteers performing two ordinary respiratory conditions (i.e., eupnea and tachypnea) in a sitting stance. The promising results suggest that the presented system could pave the way towards the easier and more comfortable monitoring of hemiplegics’ respiratory activity, both in clinical and in domestic environments.

## 2. Materials and Methods

The experimental set-up, the features of the population taking part in the study, and the experimental protocol are described in the following subsections.

### 2.1. Experimental Set-Up

**Figure 1:**
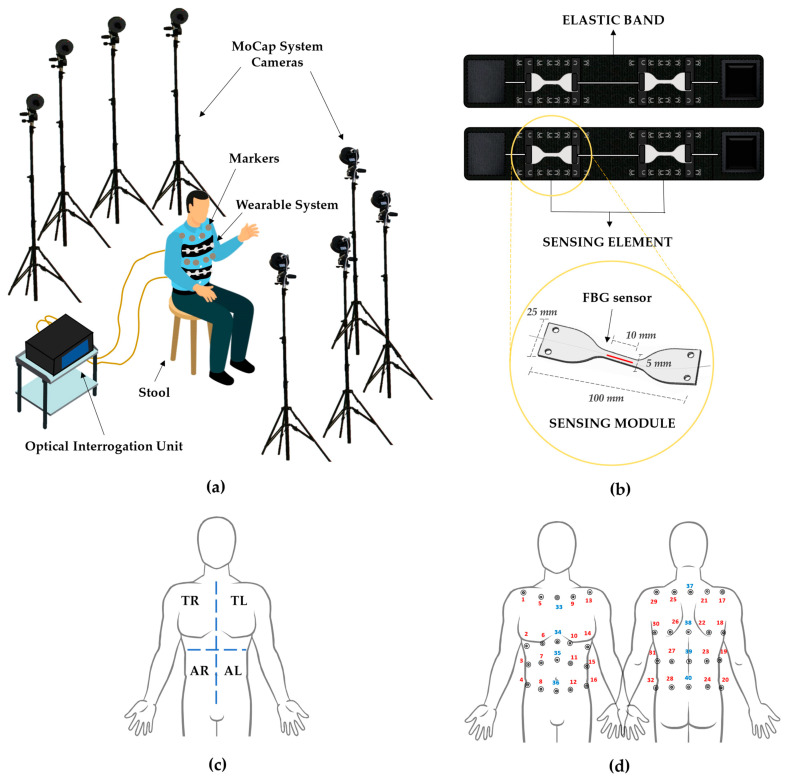
The experimental set-up, composed of a stool, the eight cameras of the motion capture system, the reflective markers, the wearable system, and the optical interrogation unit, is depicted in (**a**). In (**b**), an insight into the wearable system is given, showing the two elastic bands, each of which is equipped with a sensing element. In addition, a magnification of a sensing module is reported. In (**c**), the four macro-areas (right thorax—TR, left thorax—TL, right abdomen—AR, and left abdomen—AL) into which the torso is subdivided are displayed. In (**d**), the positioning of the 40 reflective markers on the subject’s torso is shown.

The experimental set-up depicted in Figure 1a is composed of the following elements.

1. Stool: a stool placed in the center of the dedicated area allows the volunteers to sit.
2. Motion Capture System: a stereophotogrammetric MoCap system was exploited as a reference device for chest wall kinematics and respiratory activity. The system (BTS D-Smart, produced by BTS Bio-Engineering S.r.l., Milan, Italy) consists of eight cameras, installed at approximately 2 m from the stool, four forward and four rearward, as shown in Figure 1a. An amount of 1 m^3^ of calibrated volume was obtained. The trajectories of 40 photo-reflective hemispherical markers with a diameter of 12 mm (applied to the volunteers’ torsos, as detailed in Section 2.2) were collected with a sampling rate of 60 Hz by means of the tracker software provided by BTS (BTS, Bioengineering S.r.l., Milan, Italy);
3. Wearable System: the wearable system (represented in Figure 1b, upper image) comes as two stretchable elastic bands to be worn around the chest and abdomen, to each of which a sensing element is attached by means of an anchoring system consisting of hooks to be secured to metallic loops sewed on the elastic bands. Every sensing element is composed of two multiplexed sensing modules (SMs), which come as dumbbell-shaped flexible matrices (see Figure 1b, bottom image) made of a commercial bi-phasic polymer (i.e., DragonSkin^TM^ 20, Smooth-on-Inc.) with an overall length of 100 mm, and a width of 25 mm at the edges and 5 mm in the narrow portion. Each SM encloses in its center a 10-mm-length fiber Bragg grating (FBG) sensor (Broptics Technology Inc., reflectivity >99.5%, polarization-dependent loss <0.15 dB, insertion loss <0.2 dB, and cladding mode loss <0.2 dB). FBGs are resonant structures whose length is typically in the range of millimeters, and they are located in specific sections of the fiber optic via laser beam inscription. These structures reflect to the source a narrow portion of light that is centered around a particular wavelength (i.e., Bragg wavelength—λ_B_), different for each FBG. λ_B_ meets the following condition of dependence on the effective refractive index (η_eff_) and the grating period (Λ) [38]:
  λ_B_ (ε, ΔT) = 2·η_eff_ (ε, ΔT) · Λ (ε, ΔT) (1)

As both η_eff_ and Λ are deeply influenced by strain (ε) and temperature variations (ΔT), changes in these parameters cause a shift in λ_B_ (Δλ_B_). Nevertheless, as already specified in [37], in the present scenario of interest, the contribution of ΔT to Δλ_B_ can be deemed negligible. As a consequence, Δλ_B_ values are only results of the ε variation (which are directly transmitted to the FBG via matrix–sensor bonds) provoked by the chest wall displacements related to the breathing activity. In particular, during the inspiration phase, the rib cage expands, causing the SMs to elongate until reaching maximal traction at the end of the inspiration. On the contrary, during the expiratory phase, the rib cage compresses, resulting in compression of the SMs, which is maximal at the end of the expiration.

Further information on the design and manufacturing process of the wearable system, as well as its metrological properties, can be found in [37].

4. Optical Interrogation Unit: an optical interrogation unit (si255, developed by Micron Optics Inc., Atlanta, GA, USA, wavelength range of 1460–1620 nm) was employed to interrogate the FBGs contained in the sensing modules. The interrogation unit supplies the sensors with broadband polarized light and collects the Δλ_B_ values at a 1 kHz sampling rate.

### 2.2. Population and Experimental Protocol

Seven post-stroke hemiplegic patients being treated at the Physical and Rehabilitation Unit of Fondazione Policlinico Universitario Campus Bio-Medico were recruited from the research volunteer database. The informed consent form was read and signed by all the participants. All patients fulfilled the inclusion criteria, i.e., an established post-stroke hemiplegia diagnosis and absence of cognitive limitations preventing comprehension of the experimental protocol. The features of the population that took part in the study are reported in Table 1, together with the related Fugl–Meyer Assessment Upper Extremity score [39] to quantify the upper body disability extent.

Each participant was asked to wear a tight shirt and sit on the stool. Then, the operator helped the subject to wear the wearable system. The two elastic bands were fastened around the torso and secured at the thoracic and abdominal levels with the help of some Velcro^®®®^ strips. A sensing element was positioned on each elastic band by placing one SM on the left side and one on the right side, symmetrically with respect to the longitudinal axis of the body. Therefore, considering the torso divided into the four macro-areas depicted in Figure 1c (i.e., right and left thorax—TR and TL, respectively—and right and left abdomen—AR and AL, respectively), an SM was placed in correspondence to each area. The SMs were then secured to the bands by means of the modular anchoring system. Moreover, 40 photo-reflective hemispherical markers were applied to the patient’s torso, 20 on the front and 20 on the back, as depicted in Figure 1d. The used marker protocol is an enhancement of the one presented by Ferrigno et al. [40], exploiting 32 markers for thoraco-abdominal kinematic detection and compartmental respiratory volume evaluation. Further details on the adopted marker protocol can be retrieved in Appendix A.

The volunteer was instructed on the experimental protocol, which consisted of two trials:

5. Trial 1: 5 s of apnea followed by 40 s of eupnea, maintaining the upright sitting position with the hands resting on thighs;
6. Trial 2: 5 s of apnea followed by 30 s of tachypnea (to the best of each subject’s ability), maintaining the upright sitting position, hands resting on thighs.

The design of the presented protocol was conceived to assess the ability of the wearable system to monitor the respiratory activity of the hemiplegic patients in two ordinary breathing conditions (i.e., eupnea and tachypnea). The experimental protocol was designed to meet the patients’ capacities and preserve their health status. The study protocol was approved by the Ethics Committee of Università Campus Bio-Medico di Roma (protocol code ST-UCBM 27.2(18).20 OSS), in conformity with the percepts of the Declaration of Helsinki.

## 3. Data Analysis and Results

### 3.1. Preliminary Data Processing

Data obtained by the four SMs of the wearable system and MoCap were processed in the Matlab^®®®^ environment (MathWorks^®®®^ Inc., Natick, MA, USA). Markers’ data were analyzed to retrieve the total respiratory volume (V_TOT_) and the respiratory volumes related to the four torso areas (i.e., V_TR_, V_TL_, V_AR_, and V_AL_). In particular, the chest wall volume was derived by the 3D marker coordinates by using the geometric model reported in [41]. In detail, the torso was divided into four compartments (i.e., TR, TL, AR, and AL), each of which was represented by eight markers (i.e., four markers in the front and four in the back of the corresponding area) identifying six tetrahedrons [42]. Considering P_1,i_, P_2,i_, P_3,i_, and P_4,i_, the vertices of the *i*-th tetrahedron, the *i*-th volume ($V_{i}$), can be retrieved as below:

(2) $$V_{i}=\frac{1}{6}\;\left|\det\left(V_{1,i},V_{2,i},V_{3,i}\right)\right|=\frac{1}{6}\left|det\left[\begin{array}{cccc} 1 & x_{P1} & y_{P1} & z_{P1}\\ 1 & x_{P2} & y_{P2} & z_{P2}\\ 1 & x_{P3} & y_{P3} & z_{P3}\\ 1 & x_{P4} & y_{P4} & z_{P4} \end{array}\right]\right|$$

where V_1,i_ = P_2,i_ − P_1,i_, V_2,i_ = P_3,i_ − P_2,i_, and V_3,i_ = P_4,i_ − P_3,i_. V_TOT_ was then calculated by summing the volumes of all the tetrahedrons, while the respiratory volumes of each compartment (i.e., V_TR_, V_TL_, V_AR_, and V_AL_) were calculated as the sum of the volumes belonging to the specific compartment.

Signals retrieved by the SMs placed in TR, TL, AR, and AL will be hereafter named as SM_TR,_ SM_TL,_ SM_AR_, and SM_AL_, respectively.

**Figure 2:**
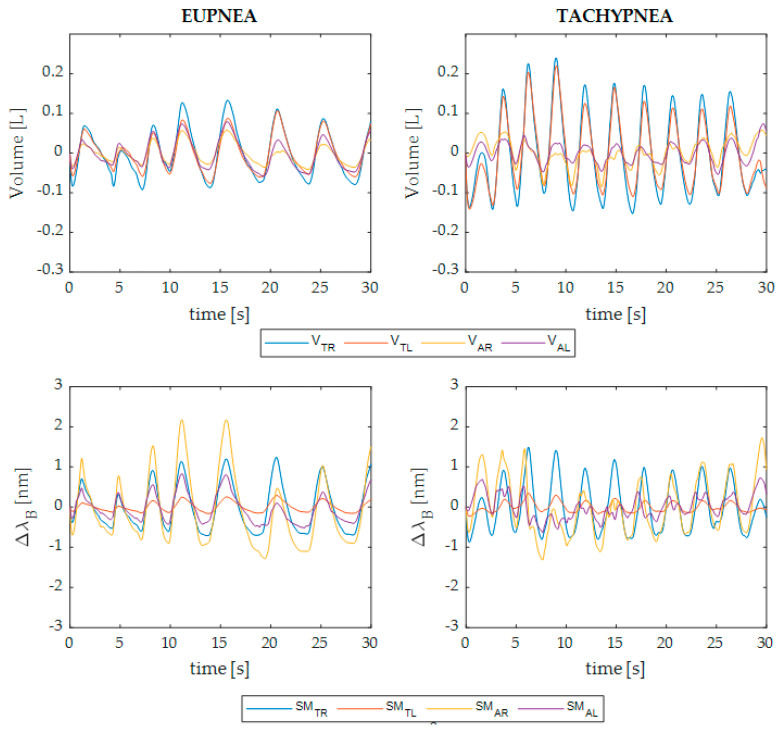
Example of the respiratory signals retrieved by the MoCap (upper graphs) and wearable system (bottom graphs) during 30 s of eupnea and tachypnea trials (left and right columns, respectively) performed by a patient. In blue, orange, yellow, and purple are shown the signals related to the TR, TL, AR, and AL compartments, respectively (i.e., V_TR_, V_TL_, V_AR_, and V_AL_ for the MoCap and SM_TR_, SM_TL_, SM_AR_, and SM_AL_ for the wearable system).

SM_TR_, SM_TL_, SM_AR_, and SM_AL_ and V_TOT_, V_TR_, V_TL_, V_AR_, and V_AL_ were synchronized by means of the first maximum peak occurring after the 5 s apnea. To eliminate information content not exclusively related to the respiratory activity, a first-order Butterworth pass-band filter was applied with a cutoff frequency of 0.01–1 Hz to eupnea signals (i.e., Trial 1) and 0.01–2 Hz to the tachypnea signals (i.e., Trial 2). An example of the data collected in eupnea and tachypnea by one of the seven patients is given in Figure 2. It is worth noting that the amplitudes of the signals obtained by the MoCap and the SMs may not be comparable. This phenomenon is due to the different tensile conditions under which the SMs are placed on the elastic bands. In particular, the more the SMs are tightened, the wider the signals’ amplitude. However, this occurrence does not affect the RR assessment.

Both for MoCap and the wearable system, the signals related to the thoracic, abdominal, plegic, non-plegic, and all four torso areas were obtained as follows:

1. Thoracic compartment signal: sum of the signals related to the TR and TL (i.e., SM_TR_ + SM_TL_ for the wearable system and V_TR_ + V_TL_ for the MoCap);
2. Abdominal compartment signal: sum of the signals related to the AR and AL (i.e., SM_AB_ + SM_AB_ for the wearable system and V_AB_ + V_AB_ for the MoCap);
3. Plegic compartment signal: sum of the thoracic and abdominal signals related to the affected side of each patient;
4. Non-plegic compartment signal: sum of the thoracic and abdominal signals related to the non-affected side of each patient;
5. Summed signal: sum of all four torso areas’ signals (i.e., SM_TR_ + SM_TL_ + SM_AB_ + SM_AB_ for the wearable system and V_TOT_ for the MoCap).

In Section 3.2, these five respiratory signals obtained in eupnea and tachypnea will be analyzed for each volunteer to assess the wearable system’s capability for RR estimation against the MoCap system. It will be also investigated which of the five signals returns lower errors in RR detection. Then, in Section 3.3, an explorative analysis will be carried out to evaluate the capability of the wearable system to detect potential respiratory asynchronies between torso compartments.

### 3.2. Assessment of the Wearable System in Respiratory Rate Estimation

For each of the five signals obtained for every volunteer in eupnea and tachypnea, RR was estimated via a breath-by-breath approach. A single breathing act is the portion of signal enclosed between two consecutive minima. The *i*-th respiratory period ($T_{ri}$) related to the *i*-th breath was retrieved as the time elapsed between two consecutive maximum peaks (expressed in s), both for the wearable system and the MoCap ($T_{ri}^{wearable}$ and $T_{ri}^{MoCap}$, respectively). Thus, the related *i*-th RR values were obtained ($RR_{i}^{wearable}$ and $RR_{i}^{MoCap}$) as the reciprocal of the respiratory periods multiplied by 60 (as expressed in breaths per minute—bpm).

**Figure 3:**
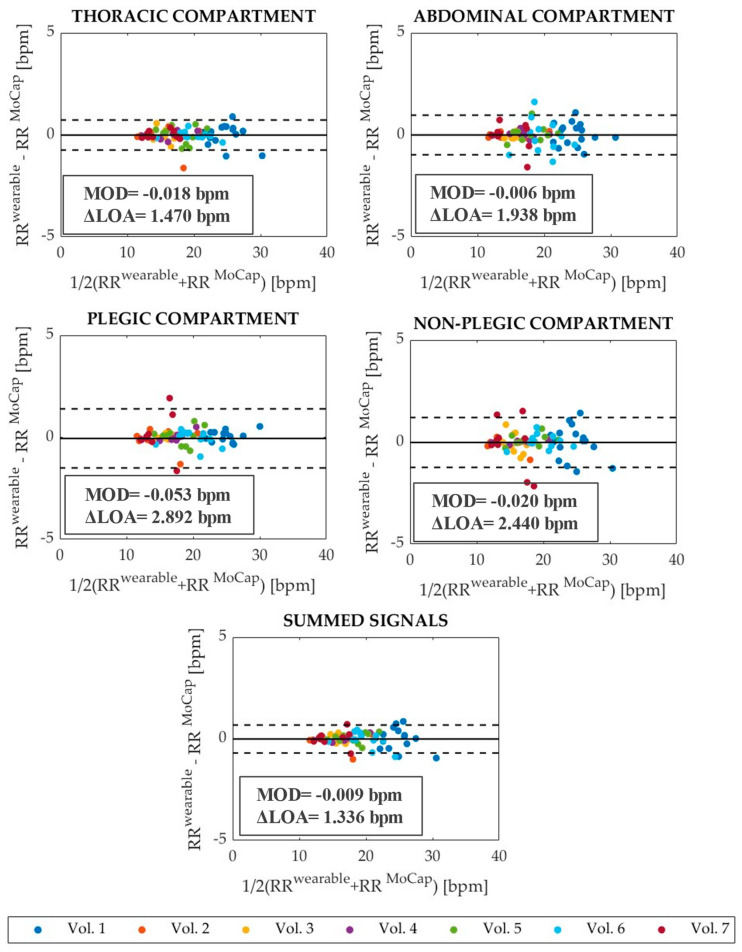
Bland–Altman plots showing the bias between $RR_{i}^{wearable}$ and $RR_{i}^{MoCap}$ calculated from the thoracic, abdominal, plegic, and non-plegic compartments’ data and the summed signals retrieved during the eupnea trials (Trials 1). The MOD and ΔLOA are reported on each graph. In addition, MOD is represented with black solid lines, while ΔLOA is the span comprised between the two black dotted lines.

**Figure 4:**
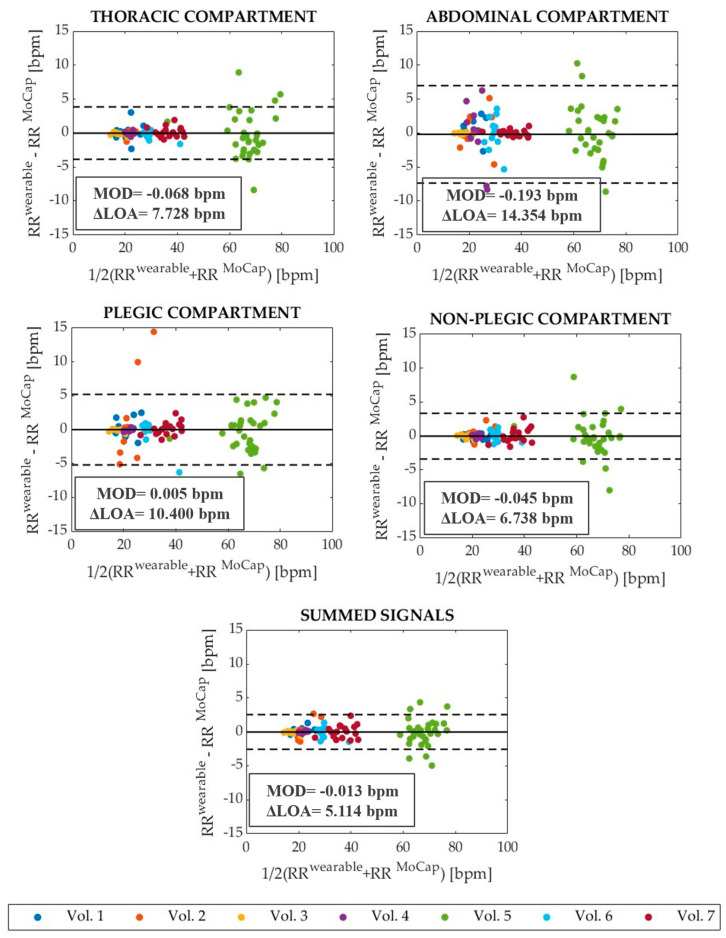
Bland–Altman plots showing the bias between $RR_{i}^{wearable}$ and $RR_{i}^{MoCap}$ calculated from the thoracic, abdominal, plegic, and non-plegic compartments’ data and the summed signals retrieved during the tachypnea trials (Trials 2). The MOD and ΔLOA are reported on each graph. In addition, MOD is represented with black solid lines, while ΔLOA is the span comprised between the two black dotted lines.

To quantify the accordance between the wearable system and the reference device in RR evaluation, Bland–Altman analysis [43] was carried out, providing the mean of differences (MOD) and the limits of agreement (LOAs) expressed as MOD ± 1.96 SD (where SD is the standard deviation). The Bland–Altman graphs obtained for the four compartments (i.e., thoracic, abdominal, plegic, and non-plegic) and summed signals related to the eupnea (Trials 1) and tachypnea (Trial 2) are shown in Figure 3 and Figure 4, respectively. MOD values and the values of the span between the LOAs (i.e., ΔLOA calculated as 2 1.96 SD) are reported on each graph. MOD values are almost comparable in all compartments and summed signals in eupnea, while they present a higher value in the abdominal compartment in tachypnea. In both eupnea and tachypnea, ΔLOAs are always lower for the summed signals.

The mean absolute percentage error (MAPE) was also calculated to determine the error committed by the wearable system in evaluating RR as

(3) $${\mathrm{MAPE}}_{\mathrm{RR}}=\frac{1}{n}\overset{n}{\underset{i=1}{\sum }}\frac{|RR_{i}^{wearable}-RR_{i}^{MoCap}|}{RR_{i}^{MoCap}}\;\cdot 100$$

where $RR_{i}^{wearable}$ and $RR_{i}^{MoCap}$ are the RR calculated for the *i*-th breathing act from the wearable system and MoCap data, respectively, and *n* is the number of respiratory acts identified in each signal. The MAPE_RR_ values related to all five analyzed signals are reported in Table 2 for every volunteer in eupnea (Trial 1) and tachypnea (Trial 2), and for all the volunteers together (considering the vectors in which the RR values of all subjects are concatenated).

In eupnea (Trial 1), the maximum MAPE among all the volunteers is reported for the non-plegic compartment (i.e., 2.08%), with MAPE values for individual subjects ranging from 0.37% (volunteer 4, non-plegic compartment) to 6.09% (volunteer 7, plegic compartment). In tachypnea (Trial 2) a general slight increase in the MAPE values is observed and the maximum value among all the volunteers is obtained for the abdominal compartment (i.e., 5.61%). Peak values of 18.55% (volunteer 2, plegic compartment) and 14.36% (volunteer 4, abdominal compartment) were achieved in two individuals due to the poor quality of the retrieved breathing signals. Moreover, the best performance was given by the summed signal in both the eupnea and tachypnoea trials, presenting values of 1.22% and 2.06% for MAPE calculated on all the volunteers, respectively. Consequently, considering that the lowest MAPE overall and the ΔLOA values retrieved from the Bland–Altman analysis were always obtained for the summed signals, it can be assumed that among the five proposed, the summed signals are the most suitable for RR estimation.

### 3.3. Explorative Investigation on Respiratory Asynchronies between Compartments

An explorative investigation via the loop technique was performed to assess the capability of the wearable system to detect the presence of phase shifts. The analysis was carried out comparing the respiratory signals of the thoracic and abdominal compartments, and plegic and non plegic-compartments, retrieved for different volunteers in the tachypnea trials.

**Figure 5:**
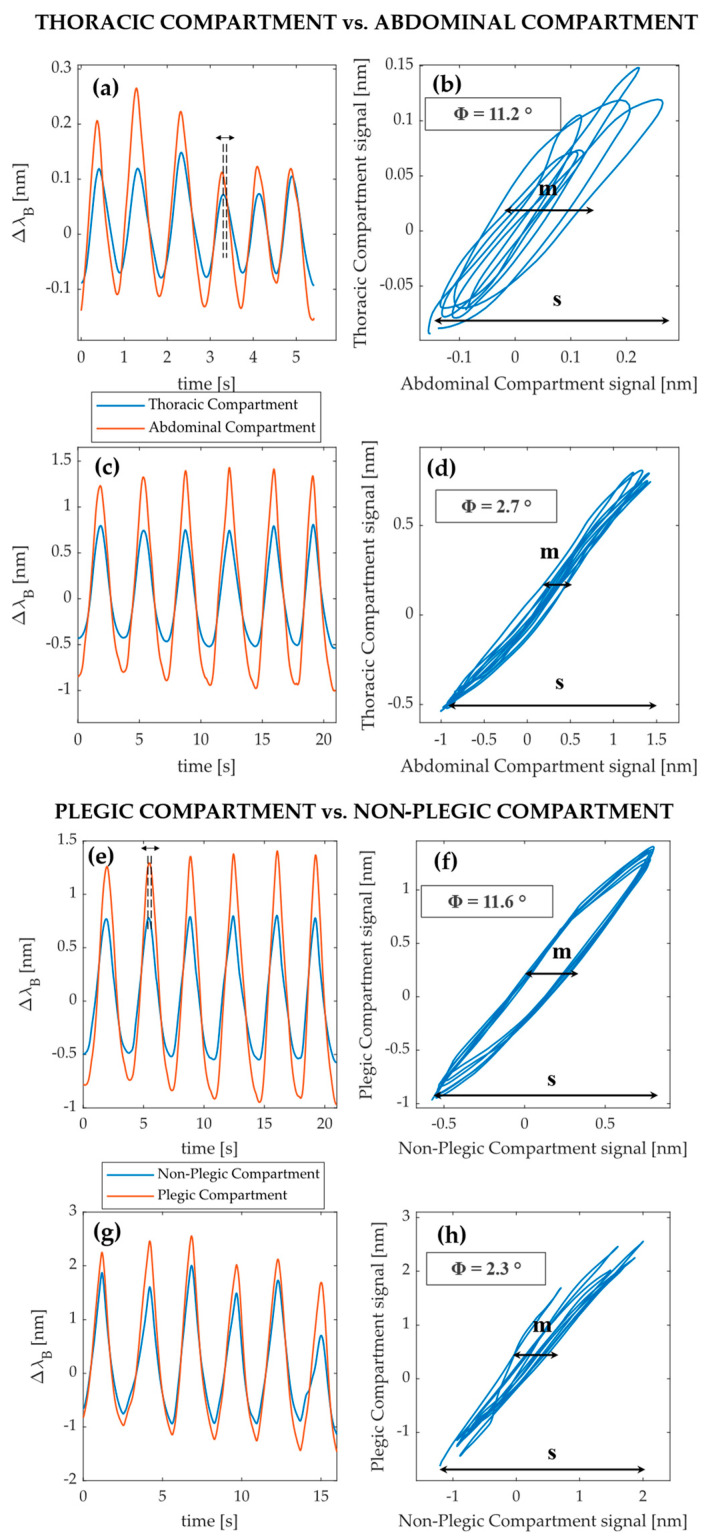
Phase shift analysis between thoracic and abdominal compartments and plegic and non-plegic compartments. Plots of thoracic and abdominal compartments’ signals in time for two different subjects are shown in (**a**,**c**). In (**a**), a phase shift is shown, while in (**c**), no significant shifts are visible. Lissajous figures of the out-of-phase (**b**) and in-phase (**d**) signals are reported, together with the related phase angle value. Plots of plegic and non-plegic compartments’ signals in time for two different subjects are reported in (**e**,**g**). In (**e**) and in (**g**), out-of-phase and in-phase signals are shown, respectively. Lissajous figures of the out-of-phase (**f**) and in-phase (**h**) signals are reported, together with the related phase angle value.

Referring to the comparison between the thoracic and abdominal compartments, the two best signals representing six consecutive and homogeneous respiratory acts were chosen among all the volunteers and were identified for volunteers 3 and 5. The signals were then plotted in time. In Figure 5a (referring to volunteer 5), it is possible to observe that the signals are out-of-phase as the thoracic anticipates the abdominal one, while in Figure 5c (referring to volunteer 3), the two signals are in-phase. The related Lissajous figures were obtained by plotting the two respiratory signals against each other (i.e., thoracic signal on the y-axis and abdominal signal on the x-axis). For each loop (which corresponds to a single respiratory act), the *Φi* was calculated as follows [8]:

(4) $$\Phi i=\sin^{-1}\left(\frac{m_{i}}{s_{i}}\right)$$

where $m_{i}$ is the maximum distance of the *i*-th loop projection on the x-axis, while $s_{i}$ is the distance of the loop projection on the x-axis at 50% of the thoracic signal. *Φ* was retrieved for each volunteer as the average value of all *Φi*.

Values of *Φ* = 11.2° (see Figure 5b) and *Φ* = 2.7° (see Figure 5d) were obtained for the first and second volunteers, respectively.

The same analysis was performed for the plegic and non-plegic compartments, choosing the signals retrieved for volunteers 3 and 4. In Figure 5e (referring to volunteer 3), out-of-phase signals are shown (i.e., the plegic precedes the non-plegic signal), while in Figure 5g (referring to volunteer 4), in-phase signals are represented. Once again, *Φ* was calculated, obtaining *Φ* = 11.6° (see Figure 5f) and *Φ* = 2.3° (see Figure 5h).

## 4. Discussion

Variations in RR may help to evaluate and monitor the general health status of hemiplegics and control the fatigue status that these patients undergo during rehabilitation sessions.

In this study, the feasibility assessment of a multi sensor FBG-based wearable system in monitoring RR in seven hemiplegic patients performing eupnea and tachypnea breathing has been performed. The wearable system, originally developed to be exploited in challenging working conditions (e.g., lifting loads, walking, etc.) [37], comes as two elastic bands equipped with two multiplexed, flexible SMs composed of a dumbbell-shaped polymer matrix embedding an FBG sensor. The system is easy and comfortable to wear, and the modular anchoring system allows it to fit to all body anthropometries. Moreover, the light weight and unobtrusiveness ensure continuous monitoring, avoiding movement limitations. This allows for easy respiratory monitoring both in clinical and domestic scenarios.

Firstly, four signals representing four torso compartments (i.e., thoracic compartment, abdominal compartment, plegic compartment, and non-plegic compartment signals) and one representing the entire torso (i.e., summed signal) were obtained from different combinations of the data acquired from each volunteer in eupnea and tachypnea. These five signals were analyzed to assess the capability of the wearable system in RR detection against MoCap via the Bland–Altman statistical method and MAPE evaluation. Good agreement between the wearable system and the reference instrument was shown. In eupnea, MOD values were comparable and close to 0 bpm, and ΔLOAs were obtained between 1.336 bpm (summed signal) and 2.892 bpm (plegic compartment). Overall, MAPE_RR_ is lower for summed signals (1.22%). MAPE_RR_ values of 1.81% and 2.08% were retrieved for plegic and non-plegic compartments, although ΔLOA is greater in the plegic than in the non-plegic compartment (i.e., 2.892 bpm vs. 2.440 bpm, respectively); however, the difference is not marked. Regarding tachypnea, slightly higher errors and wider ΔLOAs were found. The best performance in terms of MOD, ΔLOAs, and MAPE is once again given by the summed signals. Increased MAPE_RR_ values are obtained for the abdominal and plegic compartments (i.e., 5.61% and 4.30%, respectively). This can be explained considering that tachypnea is a challenging respiratory condition for hemiplegic subjects, who may have used trunk compensatory movements to sustain the breathing effort, thus increasing the committed error. However, the shown errors are always <6%, which can be comparable to those present in the literature [31]. Moreover, the errors committed in RR detection from the summed signals (i.e., 1.22% and 2.06% in eupnea and tachypnea, respectively) are in line with the ones shown in [37] (i.e., 1.6%) for the application of the same wearable system on healthy volunteers in static conditions (i.e., standing, sitting, and supine positions). This preliminary analysis shows that the best signal for RR monitoring is the summed one.

Compared to the most popular and established technologies for respiratory monitoring represented by MoCap and flowmeters, the presented wearable device provides multiple advantages in terms of wearability, encumbrance, costs, and comfort. In fact, the ease of wearing and the limited encumbrance allow for the comfortable, continuous monitoring of the patient, with no need for dedicated spaces or personnel, potentially even in domestic scenarios.

Moreover, considering the target population that the study focused on, our device presents a marked improvement in terms of fit and practicality also compared to smart T-shirts [28,30,32]. Indeed, T-shirts can be difficult to wear among individuals with limited limb motility, such as people affected by hemiplegia. Additional concerns may occur as a single size could not be fit to all the anthropometries. Moreover, the particular cleaning process required to sanitize the garment without damaging the embedded sensors may represent an additional limit in T-shirts’ usability. On the contrary, the proposed system, which consists of a belt onto which the sensing elements are attached, allows for easier dressing of the subject, while the modular anchorage system guarantees good fitting to any physicality. In addition, since the SMs are removable, the elastic bands can be cleaned following ordinary washing routines, without sensor damage. Furthermore, compared to sensors directly attached to the fabric, the FBGs’ integration into the polymer matrices ensured increased robustness and flexibility, allowing the sensing modules to easily adapt to the torso’s curves. Focusing on elastic bands, several devices designed to be worn around the chest and embedding a single sensor have been presented [26,29,33]. A further step forward has been made in [27], where two FBG-based belts were proposed for respiratory monitoring on the thoracic and abdominal compartments. Compared to these solutions, our system allows data acquisition in four areas of the torso, which is of prominent importance given the examined population. In fact, as is widely reported in the literature [4,5,6,7], hemiplegic subjects may develop paradoxical motion of the respiratory muscles, often resulting in breathing discoordination between compartments, especially under challenging respiratory conditions such as tachypnea. Consequently, should these involuntary movements worsen the summed respiratory signal, it would be possible to monitor the breathing parameters by means of one of the four different signal combinations presented in Section 3.2.

Such a system may also be a useful instrument to investigate more in depth the occurrence of breathing discoordination between compartments, which could result in a phase shift in the related breathing signals. As an example, the presence of phase shifts between the thoracic and abdominal compartments has been investigated by analyzing the related tachypnea signals of two different volunteers. The thoracic and abdominal compartments showed phase angles of *Φ* = 11.2° (see Figure 5b) and *Φ* = 2.7° (see Figure 5d) for the first and second volunteer, respectively. Values of *Φ* = 11.6° (see Figure 5f) and *Φ* = 2.3° (see Figure 5h) were obtained between the plegic and non-plegic compartments, respectively. Considering that *Φ* = 0° defines synchronous signals while *Φ* = 180° indicates completely asynchronous ones [8,9], such results suggest that very slight asynchronies between compartments were observed given the mild degree of hemiplegia found in the volunteers. Moreover, in one of the two patients, asynchronies were more pronounced. In addition, positive values of *Φ* mean that the thoracic and plegic compartments (whose signals were placed on the y-axis of the Lissajous figures) always preceded the abdominal and non-plegic compartments in each respiratory act.

Encouraged by these promising results, further efforts will be dedicated to expanding the number of study participants, also including volunteers with higher Fugl–Meyer indices, so as to achieve a better and more realistic representation of the hemiplegic population. Moreover, the assessment of the wearable system will be performed in more challenging scenarios (such as during rehabilitation procedures involving object handling and lifting or walking tasks) and its performance will be compared to that of other existing and more commonly used technologies. Moreover, a deeper analysis of the phase shift between compartments will be addressed, also testing the wearable system on a larger number of volunteers and with a higher level of muscular impairment.

## 5. Conclusions

In conclusion, in this study, a wearable system composed of four flexible sensing modules based on FBG technology was proposed for the respiratory monitoring of hemiplegic patients. The device feasibility assessment in RR evaluation in eupnea and tachypnea breathing was performed against a reference instrument on seven hemiplegic volunteers, showing good performance. An error <6% was obtained in RR detection. Further strengths of the present device lie in its design, which allows easy fitting and comfortable application to patients, ensuring no movement limitation or chest restrictions. Moreover, the wearable system’s sensing module redundancy permits the collection of data on breathing-related chest wall deformations in four torso areas. This is particularly relevant considering that, under challenging respiratory conditions (e.g., tachypnea), hemiplegics may display paradoxical activation of the impaired torso musculature, which could worsen the signals and cause artefacts.

The main limitation of the proposed wearable system lies in the need for an external interrogation unit to collect the sensors’ data. However, considering the device’s application in clinical and domestic rehabilitation scenarios, this could represent a limited drawback. Nevertheless, the growing need for portability has stimulated the development of ever smaller and more portable interrogators, which are becoming increasingly present on the market.

The presented wearable device may be a useful tool to assist clinicians and operators in evaluating the general health status of hemiplegic patients and tracking their evolution in time. Moreover, this device could help in investigating the phenomenon of phase shift between torso compartments.

## Figures and Tables

**Table 1:** Features of the enrolled hemiplegic volunteers.

# Volunteer	Age [y.o.]	Sex	Affected Side	BMI [kg·m^−2^]	UE-FMA ^1^
1	73	Male	Left	34.6	37
2	62	Male	Left	26.7	32
3	46	Female	Right	19.3	33
4	64	Male	Right	32.7	43
5	33	Female	Left	19.1	55
6	55	Male	Right	17.4	50
7	43	Male	Left	27.6	34

^1^ Fugl–Meyer Assessment Upper Extremity score.

**Table 2:** MAPE_RR_ [%] values reported for every volunteer in eupnea (Trial 1) and tachypnea (Trial 2).

**Trial 1—EUPNEA**					
**# Volunteer**	**MAPE_RR_ [%]** **Thoracic**	**MAPE_RR_ [%]** **Abdominal**	**MAPE_RR_ [%]** **Plegic**	**MAPE_RR_ [%]** **Non-Plegic**	**MAPE_RR_ [%]** **Summed**
1	1.51	1.93	0.86	2.57	1.60
2	1.78	0.72	1.91	1.28	1.15
3	1.47	0.78	0.62	2.79	1.08
4	0.69	0.69	0.79	0.37	0.52
5	2.03	1.58	1.87	1.11	1.08
6	0.73	3.45	1.46	1.35	1.34
7	1.07	2.66	6.09	5.23	1.52
All	1.32	1.77	1.81	2.08	1.22
**Trial 2—TACHYPNEA**					
**# Volunteer**	**MAPE_RR_ [%]** **Thoracic**	**MAPE_RR_ [%]** **Abdominal**	**MAPE_RR_ [%]** **Plegic**	**MAPE_RR_ [%]** **Non-Plegic**	**MAPE_RR_ [%]** **Summed**
1	4.25	5.76	5.68	1.91	2.06
2	1.31	8.63	18.55	3.33	4.41
3	1.08	0.89	0.87	1.13	1.00
4	0.97	14.36	1.05	1.04	0.52
5	3.93	5.60	3.38	2.97	2.18
6	1.70	5.46	2.44	2.17	1.94
7	1.39	0.97	1.87	2.22	1.94
All	2.45	5.61	4.30	2.33	2.06

## Data Availability

The data presented in this study are available on request from the corresponding author. The data are not publicly available due to privacy reasons.

## Appendix A

To calculate the patients’ compartmental breathing volumes starting from the chest wall kinematic data, different algorithms exploiting diverse marker protocols (i.e., based on 89, 86, 32, and 30 markers) have been presented in the literature [41]. However, while protocols requiring a large number of markers present evident drawbacks related to time consumption and limited reproducibility, the 32-marker-based protocol proposed in [40] was demonstrated to be sufficiently accurate in the respiratory volume detection of the thoracic and abdominal compartments. Starting from this, in this work, we adopted a 40-marker protocol to better differentiate the left and right sides of the chest wall and evaluate the related respiratory volumes.
